# Saccharin consumption increases sperm DNA fragmentation and apoptosis in mice

**Published:** 2014-05

**Authors:** Marzieh Rahimipour, Ali Reza Talebi, Morteza Anvari, Abolghasem Abbasi Sarcheshmeh, Marjan Omidi

**Affiliations:** 1 *Department of Biology and Anatomical Sciences, Shahid Sadoughi University of Medical Sciences, Yazd, Iran.*; 2 *Department of Andrology, Research and Clinical Center for Infertility, Shahid Sadoughi University of Medical Sciences, Yazd, Iran.*

**Keywords:** *Sperm*, *Saccharin*, *Apoptosis*, *Mice*

## Abstract

**Background:** Saccharin is an artificial non-caloric sweetener that used to sweeten products such as drinks, candies, medicines, and toothpaste, but our bodies cannot metabolize it. Sodium saccharin is considered as an important factor in tumor promotion in male rats but not in humans.

**Objective:** The objective of this study was to investigate the effect of saccharin consumption on sperm parameters and apoptosis in adult mice.

**Materials and Methods: **Totally 14 adult male mice were divided into 2 groups. Group 1 served as control fed on basal diet and group 2 or experimental animals received distilled water containing saccharin (0.2% w/v) for 35 days. After that, the left cauda epididymis of each mouse was cut and placed in Ham’s F10. Swimmed-out spermatozoa were used to analyze count, motility, morphology (Pap-staining) and viability (eosin-Y staining). Sperm DNA integrity, as an indicator of apoptosis, was assessed by SCD (sperm chromatin dispersion) and terminal deoxynucleotidyl transferase (TUNEL) assay.

**Results: **Following saccharin consumption, we had a reduction in sperm motility with respect to control animals (p=0.000). In addition, the sperm count diminished (17.70±1.11 in controls vs. 12.80±2.79 in case group, p=0.003) and the rate of sperm normal morphology decreased from 77.00±6.40 in control animals into 63.85±6.81 in saccharin-treated mice (p=0.001). Also, we saw a statistically significant increase in rates of sperm DNA damage and apoptosis in experimental group when compared to control one (p=0.001, p=0.002 respectively).

**Conclusion:** Saccharin consumption may have negative effects on sperm parameters, and increases the rate of sperm DNA fragmentation and apoptosis in mice.

## Introduction

Saccharin is the first artificial and non-caloric sweetener which was synthesized in 1879. Since, it is significantly more potent than sucrose; just small amounts are needed to sweeten foods (1, 2). Saccharin is commercially available in acid form as well as in sodium and calcium salts forms. The most commonly used form of saccharin is the sodium salt. All of these are white odorless solids. Saccharin in its acid form is poorly soluble in water while its salt forms are highly soluble (1). 

Over the last century, saccharin and its salts have been used in a variety of beverages, foods, cosmetics and pharmaceuticals whereas; our bodies cannot metabolize saccharin (2, 3). It is used in the following foods and beverages: soft drinks, fruit juices, other beverages and other beverage bases or mixes; powder or liquid form; processed fruits, chewing-gum and confections; gelatin desserts, jams, sauces and dressings. Lesser amounts of saccharin are used in a variety of non-food applications, as a nickel electroplating brightener, chemical intermediate, animal feed sweetener and anaerobic adhesive accelerator (3). The carcinogenicity of saccharin has been the subject of numerous animal and epidemiologic studies. Results from standard long-term rodent carcinogenicity tests indicate that sodium saccharin is a weak carcinogen and tumor promoter in the male rat bladder. Probably, the mechanism for saccharin induction of bladder tumors in rats is specific to the physiology of the male rat bladder (4). The absence of any certain epidemiologic data states that saccharin is associated with increased tumors in human has led to sodium saccharin being classified as a non-carcinogen compound in human (5).

Furthermore, some of the studies showed that saccharin consumption induces cellular and cytogenetic changes. For example, Chinese hamster ovary cells and human lymphocytes were exposed to the sodium saccharin by Wolff S and Rodin B. They revealed that saccharin and extremely purified extract of it, increased the yield of sister chromatid exchanges in both types of cells. So, in addition to be a weak carcinogen in rat, it is also mutagenic and moderately induces cytogenetic changes (6).

Apoptosis is a physiological process of programmed cell death that occurs throughout the body and it is controlled by different regulatory elements in different tissues (7, 8). Morphologically, apoptosis is characterized by nuclear chromatin condensation, cell shrinkage, apoptotic body formation and DNA fragmentation (9). In the testis, apoptosis is controlled by Sertoli cells through the “apoptosis stimulating fragment” or Fas system characterized by the interaction between Fas ligand expressed by Sertoli cells and Fas receptors expressed by the germ cells (8, 10). After the binding of Fas ligands, the activation of some particular proteinases called caspases will be occurred (10). 

The activation of caspase-3 as the most effective caspase, results in the activation of Ca/Mg dependent endonucleases that break DNA into nucleosomal units with 185 bp (11, 12). It has been shown that any defects in the remodeling of cytoplasm during spermatogenesis and presence of cytoplasmic retentions may lead “abortive apoptosis” a phenomenon in which defective sperm cells escape programmed cell death and are seen in the ejaculate (13). The sperm DNA integrity evaluations may be a better device than routine semen analysis in male fertility assessments (14). It should be noted that in addition to infertile men with abnormal semen parameters, 8% of men with normal sperm indices, different forms of sperm DNA damage are found that lead to infertility (15). 

So the objective of this study was to examine the possible detrimental effects of saccharin on sperm parameters and apoptosis in adult mice.

## Materials and methods

**Animals and experimental design**

In this experimental study, fourteen 10 weeks adult male mice with an average weight of 30g were housed in clean cages in an air-conditioned room under controlled temperature (25±3^o^C), suitable humidity (50±5%) and a 12h light/dark cycle. Animals were randomly divided into 2 groups (n=7). The first group served as control allowed free access to mouse food and water but, group 2 or experimental group, was given ad libitum access to water containing saccharin (0.2% w/v; Fluka, Switzerland) for 35 days. This experimental project was approved by ethical committee of Shahid Sadoughi University of Medical Sciences.

**Epididymal sperm preparation**

After 35 days (more than one duration of spermatogenesis in mice), a small piece of the cauda epididymis of each animal was dissected and placed in 1 mL of pre-warmed Ham’s F10 medium (37^o^C, 5% Co_2_). The tissue was cut quietly to make spermatozoa swim-out into the culture medium and was located in the incubator for 15 min.

**Sperm analysis**

For 200 spermatozoa of each animal, sperm parameters including count (10^6^/ml), motility (%), viability and normal morphology (%) were evaluated. We used Makler chamber (Sefi Medical Co., Haifa, Israel) to access sperm count and motility (16). Motility was expressed as percentage of progressive (rapid and slow) and non-progressive spermatozoa. Eosin test and Papanicolaou staining for evaluating sperm viability and morphology were used respectively (17, 18). 

**Evaluation of sperm apoptosis**

**SCD test**

To determine DNA fragmentation rates in semen samples, we used the Sperm Chromatin Dispersion (SCD) test according to Fernandez *et al* protocol (19). In this test, the glass slides were coated by 0.65% standard agarose (Merck, Germany) and 30 µL of sperm suspension was mixed with 70 µL low melting agarose. Then Aliquots of 50 µL of the mixture were put onto pre-coated glassy slides and were left to solidify at 4^o^C for 4 min. The slides were immersed in denaturation solution (0.08 NHCl) (Merck, Germany) for 17 min at room temperature in darkness. Then the slides were transferred into lysing solution 1 (0.4 M Tris, 0.8 M 2-Mercaptoethanol, 1% SDS and 50 mM EDTA, pH=7.5), lysing solution 2 (0.4 M Tris, 2 M NaCl and 1% SDS, pH=7.5) and finally Trisborate-EDTA buffer (0.09 M Tris-borate and 0.002 M EDTA, pH 7.5) for 20, 15 and 12 min respectively. For dehydration, the samples were placed in 70%, 90% and 100% ethanol. 

In staining step, the slides were covered by wright (wright stain+PBS, 1:1) for 10 min followed by washing in tap water (19). Finally, 200 spermatozoa were evaluated by means of light microscopy. Spermatozoa with no DNA fragmentation show big or medium halos but spermatozoa with DNA fragmentation show a small halo.

**TUNEL assay**

The terminal deoxynucleotidyl transferase-mediated (TdT) deoxyuridine triphosphate (dUTP) nick end labeling assay (TUNEL) was used to evaluate DNA fragmentation (14). At first, smears of sperm cells were fixed in methanol for 30 min at room temperature and then the slides were washed in phosphate-buffered saline (PBS), pH=7.4. The permeabilization was done by 0.1% Triton X-100 (Merck, Germany) and 0.1% sodium citrate for 2 min on ice. After washing once with PBS, 30 µL of TUNEL mixture (Roche, USA) was added to each sample and incubated 60 min at 37^o^C in a moist chamber in the darkness. Finally, slides were washed three times with PBS and analyzed with fluorescence microscope (Olympus Co., Tokyo, Japan) (20). The nuclei of sperm cells with fragmented DNA (TUNEL^+^) showed bright green color, whereas the nuclei of the normal cells (TUNEL^-^) were seen pale green.

**Statistical analysis**

Statistical analysis was performed using the SPSS 18 software (SPSS Inc., Chicago, IL, USA). Differences between variables with normal distribution were analyzed applying ANOVA test and between groups were assessed applying Post Hoc Tests. The term ‘statistically significant’ was used to signify a two-sided p≤0.05 for sperm parameters and special tests.

## Results

**Figure 1:**
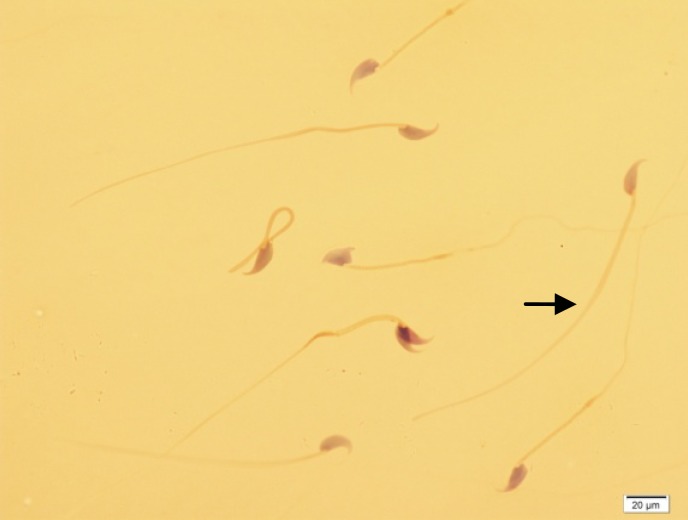
Papanicolaou staining. Different forms of sperm morphological abnormalities. The arrow indicates a normal spermatozoon. (×100 eyepiece magnification).

**Figure 2:**
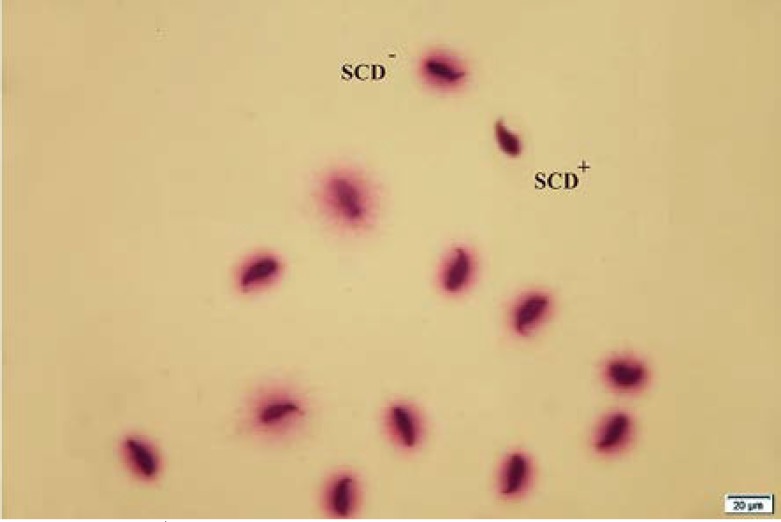
SCD test of mice spermatozoa. SCD^+^ indicates apoptotic spermatozoa and SCD^-^ indicates non apoptotic spermatozoa (×100 eyepiece magnification).

**Figure 3:**
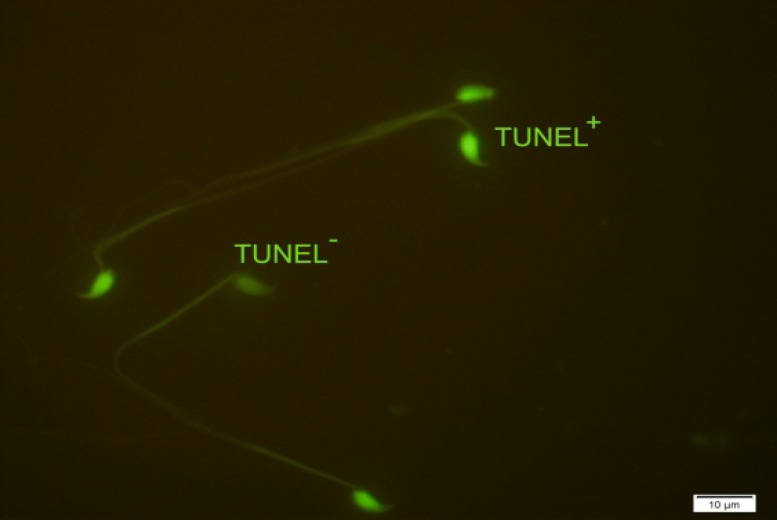
TUNEL assay of mice spermatozoa. TUNEL^+^ indicates apoptotic spermatozoa and TUNEL^-^ indicates non apoptotic spermatozoa (×100 eyepiece magnification).

As shown in table I, in control group, 74% of spermatozoa were motile whereas the total motility rate was 57% in experimental group (p=0.000). In addition to total motility, the spermatozoa of saccharin-treated and control animals revealed significantly difference in quick and slow motility (p=0.000). Moreover, there was a considerable difference between two groups in relation to viability (p=0.002) and normal morphology (p=0.001; Figure 1). Regarding to sperm count, the difference between two groups was also significant (p=0.003). Whereas, the experimental group didn’t show any detectable alteration in percentage of spermatozoa with non-progressive motility when compared to controls (p=0.912). The results of sperm chromatin staining and apoptosis are listed in table I. As it is shown in table I, the rates of spermatozoa with DNA fragmentation and apoptosis (Figures 2 and 3) were statistically higher in saccharin group than to the control one (p<0.05).

**Table I:** Effect of saccharin on sperm parameters, DNA integrity and apoptosis in mice

**Variables**	**Control group**	**Saccharin group**	**p-value**
Count (×10^6^)	17.70 ± 1.11	12.80 ± 2.79	0.003
Rapid motility (%) (Grade a)	19.28 ± 4.53	9.57 ± 4.57	0.000
Slow motility (%) (Grade b)	21.28 ± 2.87	11.85 ± 3.13	0.000
Non progressive motility (%) (Grade c)	33.28 ± 5.12	35.57 ± 7.48	0.912
Immotile sperm (%) (Grade d)	26.14 ± 3.43	43.00 ± 8.08	0.000
Total motility (%) (Grade a, b, c)	73.85 ± 3.43	57.00 ± 8.08	0.000
Normal morphology (%)	77.00 ± 6.40	63.85 ± 6.81	0.001
Viability (%)	78.85 ± 5.36	60.71 ± 6.70	0.002
TUNEL	6.57 ± 282	42.85 ± 6.76	0.001
SCD	10.00 ± 3.74	47.00 ± 6.60	0.002

Post Hoc Test.

N= 7.

Data are presented as mean±SD.

## Discussion

Since saccharin discovery, the use of this material as an artificial and non-nutritive sweetener has been the center of numerous controversies concerning to its potential toxic effects. Although, there is no data on carcinogenic effect of saccharin in mice, hamsters, monkeys and human, the sodium saccharin can induce urinary bladder tumours in male rats through formation of a urinary precipitate causing erosion of the bladder surface and extensive regenerative hyperplasia (   21 , 22). To our knowledge, there is no document indicating the detrimental effects of saccharin on sperm parameters and DNA integrity. In this study, although the saccharin consumption did not alter the rate of non-progressive motility, but the sperm progressive motility (grade a+b), total motility, count and viability decreased significantly in saccharin-treated group when compared to control one. Moreover, the results showed that the rates of sperm morphological abnormalities increased in experimental group considerably. We also assessed the sperm apoptosis, using two different tests including TUNEL assay and SCD test. 

Our data showed a relationship between saccharin consumption and sperm DNA fragmentation and the rate of sperm apoptosis in mice. To compare our results with others, though there is no specific study on saccharin, but about other sweeteners we can see some controversial data. Sariözkan *et al* showed that adding raffinose, trehalose and fructose (three different sugars) to Ham's F10 medium can provide a better protection of sperm functional parameters and DNA integrity against chilling injury in rat (23). Also, Corcuera *et al* demonstrated that freezing boar spermatozoa in extenders with increasing different concentrations of lactose adversely affected motility but provided a protective effect on acrosome (24). 

Increased lactose concentration induced higher chromatin condensation but maintained the same stability. The ability of sucrose to protect spermatozoa against DNA fragmentation during ultra-rapid cryopreservation in canine sperm was investigated by Sánchez *et al *(25). They concluded that sucrose can effectively preserve DNA integrity during ultra-rapid cryopreservation. However, the potential toxic effects of artificial sweeteners especially saccharin on the other parts of body has been examined. For instance, Dasgupta *et al *evaluated the effects of three artificial sweeteners, acesulfame K, aspartame and sodium saccharin on the contractile response of isolated rat detrusor muscle (26). Finally, they proposed that low concentrations of these artificial sweeteners enhanced detrusor muscle contraction via increased extracellular Ca^2+^ influx. They were consisting of pleomorphic microvill with variations of form, length, diameter and curvature. 

Additionally, Andreatta *et al *in a case-control study on 197 patients and 397 controls evaluated the effects of the habitual use of the most common artificial sweeteners (AS) on urinary tract in Argentina (27). They showed that the regular use of AS for 10 years or more was positively associated with urinary tract tumors. It is generally accepted that beside to semen parameters, sperm DNA damage can affect the outcome of fertility as well (13). There are 3 main causes of sperm DNA damages including abnormal chromatin packaging during spermiogenesis, abortive apoptosis and excessive production of reactive oxygen species (ROS) (28). Moreover, oxygen radicals are involved in the tumor promotion process (29). 

As the studies show, saccharin enhances ROS production in some cells. For example, Al-Saleh *et al* revealed that saccharin increases ROS production at basal and maximal glucose levels in pancreatic beta cells in a dose-dependent manner (30). So, the alterations observed in this study may be due to enhancement of ROS production in saccharin-treated mice and the ROS in form of oxidative stress are the major cause of sperm DNA damages. Even though, there is no data on the effects of saccharin on reproductive performances especially sperm fertility potential, we recommend the limited use of saccharin to diminish the possible harmful effects.

## Conclusion

The present study showed that the saccharin consumption increases the rate of sperm DNA fragmentation and apoptosis in mouse as an experimental animal. Furthermore, we showed that saccharin adversely affected sperm parameters.
